# The Illusion of Absence in Magic Tricks

**DOI:** 10.1177/2041669520928383

**Published:** 2020-06-25

**Authors:** Mats Svalebjørg, Heidi Øhrn, Vebjørn Ekroll

**Affiliations:** Department of Psychosocial Science, University of Bergen

**Keywords:** illusion of absence, amodal absence, amodal completion, attention, attentional misdirection, magic, cognitive impenetrability

## Abstract

Recently, a curious illusion of absence has been described, where the space behind an occluder is compellingly experienced as empty. This illusion is similar to illusions based on amodal completion in the sense that it refers to occluded portions of a visual scene and informal observations suggest that it may also be largely impervious to conscious knowledge. The aim of the present experiment was to test the hypothesis that the illusion of absence is cognitively impenetrable in the same way as amodal completion. Participants viewed magic tricks based on amodal completion, the illusion of absence, or attentional and reasoning misdirection and tried to infer the secret behind the tricks after one, two, or three presentations. The results show that the tricks based on the illusion of absence are very difficult to debunk, even after repeated presentations. In this regard, they are similar to tricks based on amodal completion but different from tricks based on attentional and reasoning misdirection. The participants also rated how magical they felt the tricks were. Surprisingly, the magic ratings tended to be quite high even in trials where the participants had already discovered the secret behind the trick. This unexpected finding may be taken to suggest that there may be two magical moments in the lifetime of a magic trick: In addition to the magical experience evoked by trick itself, discovering the secret behind the trick may also evoke an experience of impossibility.

Magicians often create illusions where objects seemingly appear out of (or disappear into) thin air. These illusions usually have simple explanations in which the object is hidden in a location close to where the illusion took place. For an example, take a look at Movie 1. Where do you think the apparently vanishing cigarette went? Most people will probably have a hard time figuring out the secret behind this powerful trick, although it is disappointingly simple: The cigarette was simply hidden behind the magician’s middle finger. This raises the question of why it is so difficult to figure out this very simple secret. Ekroll et al. (2017) have argued that many magic tricks, including this one, evoke a powerful perceptual illusion of absence (“amodal absence”), where the space behind an object in the foreground (in this case the middle finger) is automatically and compellingly experienced as empty. This illusion of absence is nicely demonstrated in Figure 1A and B. Arguably, the notion of such a perceptual illusion where the space behind a finger may be automatically and compellingly experienced as empty makes it more readily understandable that people have trouble figuring out the secret behind the above trick: Due to the powerful illusion, they fail to consider the possibility that something could actually be hidden behind the finger.

**Movie 1.** A magic trick based on the illusion of absence.

**Figure 1.**
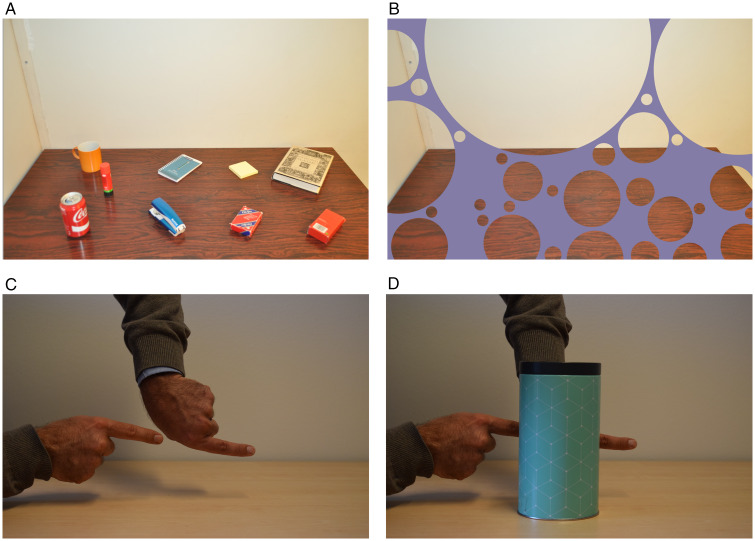
Top panels: Demonstration of the illusion of absence. Note how difficult it is to imagine that the objects on the table (Panel A) are really hidden behind the violet “bubbled” occluder in Panel B. Bottom panels: Demonstration of amodal completion. The two aligned fingers in Panel C are compellingly experienced as a single long finger when they are partially occluded by the box (Panel D). Importantly, this strong illusory impression is experienced even when you know that there are just two normal-length fingers. Top row adapted from Ekroll et al. (2017, p. 98). Copyright (2017) by SAGE Publications. Reprinted with permission. Bottom row adapted from Ekroll, De Bruyckere, et al. (2018, p. 3), used under CC BY.

The illusion of absence is reminiscent of the well-known phenomenon of amodal completion (Figure 1C and D) because it also refers to a curiously compelling experience of occluded regions in a visual scene. Amodal completion refers to experiences of complete objects partially hidden behind an occluder, which are particularly compelling although only a few fragments of the object are directly visible (Kanizsa, 1985; Michotte et al., 1964; Scherzer & Faul, 2019; Thielen et al., 2019; Van Lier & Gerbino, 2015). Figure 1D, for instance, evokes a very compelling experience of a single long finger, rather than the two separate fingers, which are aligned behind the cylinder (Figure 1C). The phenomenon of amodal completion is of great theoretical interest to perception science because it challenges naive intuitions about the distinction between seeing and thinking. As it refers to occluded objects it would seem weird to categorize it as a visual phenomenon, but on the other hand, it is very similar to visual phenomena in other regards. In particular, as emphasized by several researchers (Ekroll, Mertens, et al., 2018; Ekroll et al., 2016; Gerbino & Zabai, 2003; Kanizsa, 1985; Michotte et al., 1964), illusions based on amodal completion tend to persist in spite of conflicting conscious knowledge, in the same way as visual illusions at large. That is, they are cognitively impenetrable (Firestone & Scholl, 2016; Pylyshyn, 1999).

In all models of amodal completion, visible fragments in the visual input form the basis for some kind of perceptual extrapolation or “completion.” In the illusion of absence, however, there are no visible fragments that could function as “input” to these completion mechanisms. Thus, although the illusion of absence is reminiscent of amodal completion, it is not readily explained within the same explanatory framework. One potential explanation for the illusion of absence appeals to the principle of generic views (Albert, 2001; Albert & Hoffman, 2000; Barnhart, 2010; Freeman, 1992; Koenderink & van Doorn, 1986). According to this principle, the visual system avoids interpretations where small changes in viewing direction would produce qualitative (topological) changes in the visual input. Particularly for a small (or narrow) object in the foreground, this should make the visual system avoid interpretations where anything is hidden behind it (Ekroll et al., 2017; Øhrn et al., 2019).

The aim of this study was to investigate whether the illusion of absence is comparable to amodal completion in the sense that it is driven by perceptual, cognitively impenetrable mechanisms (Pylyshyn, 1999) as suggested by Ekroll et al. (2017). As argued by Ekroll, De Bruyckere, et al. (2018), magic tricks may be expected to be very hard to debunk even after repeated presentations, if they are based on cognitively impenetrable visual illusions. In line with the notion that amodal completion is cognitively impenetrable, they found that magic tricks based on it were indeed very difficult to debunk even after repeated presentations. They also investigated tricks based on various forms of attentional misdirection, and here repeated presentations tended to make it much easier for the spectators to figure out the secret behind the tricks. In this study, we replicated this experiment and also included tricks based on the illusion of absence. Based on the hypothesis that the illusion of absence is driven by cognitively impenetrable perceptual mechanisms, we predicted that also tricks based on this illusion should be very difficult to debunk after repeated presentations. To anticipate, our results suggest this is indeed the case.

Intuitively, it seems natural to assume that whenever the spectator is aware of the secret behind a trick, she or he will not experience it as magical. Ekroll et al. (2017), however, have argued that tricks based on cognitively impenetrable perceptual illusions may retain a certain residual magical quality even after the secret is known because perceptual illusions tend to persist even in the face of better knowledge. To elucidate this issue, we also asked the participants to rate how magical they thought the trick was at each presentation. For the tricks based on the illusion of absence and amodal completion, which by our hypothesis depend on cognitively impenetrable perceptual mechanism (in contrast to the tricks based on attentional and reasoning misdirection, henceforth “AR” tricks), we expected that the magic ratings would depend less on whether the participant had figured out the secret or not. Surprisingly, though, we found that the magic ratings for these two types of trick did not decline at all after the participant had figured out the secrets. Also surprisingly, we found that the decline after solution for AR tricks was relatively modest. We offer potential explanations for this surprising finding in the discussion.

## Methods

We performed an online experiment where the participants viewed movie clips of three kinds of magic tricks, based on the illusion of absence, amodal completion, or attentional and reasoning misdirection (“AR”). To examine how the probability that the spectators are able to figure out the secret behind the trick changes with repeated presentations, each of the nine tricks we used (three for each type, see Table 1 and the supplemental movies listed there) was presented 3 times. After each presentation, the participants were asked to indicate (a) how magical they thought the trick was (on a scale from 0 to 10) and (b) how they thought the magic trick was performed. To control for previous knowledge about the secret behind the tricks, the participants were asked—after the last presentation of each trick—to indicate whether they knew the trick from before.

**Table 1.** Description of the Tricks Used in the Experiment.

Trick label	Trick name	Spectator experience	Explanation	Underlying psychological principle (tentative)
Absence 1	Production of jumbo coin from purse	A large coin is taken out of a purse too small for the coin (Supplemental Movie 2)	The magician hides the jumbo coin in his palms and simulates taking it out from the purse	Illusion of absence
Absence 2	Disappearing cigarette (Clark & Henry, 1978)	A cigarette vanishes from the magician’s hand (Movie 1)	False transfer of the cigarette. The cigarette is palmed	Illusion of absence
Absence 3	Vanishing cards (Green & Stone, 1995)	Multiple playing cards vanish as they are dealt to the table (Supplemental Movie 3)	The cards are hidden in the hand that is “dealing” the cards	Illusion of absence
Completion 1	Rope trick (Tabary, 2004)	The ends of a rope are removed—leaving a closed loop, before reattaching the ends (Supplemental Movie 4)	The magician has a long rope and a short one. The long rope is held as a loop and the short rope is the ends removed	Amodal completion
Completion 2	Pencil through banknote (Wenk, 1986)	A pencil penetrates a bill. (Supplemental Movie 5)	The pencil is behind the bill, but has a small plastic piece that makes it appear as the pencil is inside the bill	Amodal completion
Completion 3	Linking rings (Tarbell et al., 1971)	Two metal rings are linked together (Supplemental Movie 6)	One of the rings has a small opening that makes them pass through each other	Amodal completion
AR 1	Disappearing ball	Two balls are hit against each other and become one ball (Supplemental Movie 7)	The ball that vanishes is thrown in the lap as the balls are hit against each other	Attention, repetitions
AR 2	Coin under card (Gea, 2005)	A coin vanishes and appears under a playing card (Supplemental Movie 8)	The coin is slid under the card as it is “picked up”	Attention, feigning action
AR 3	Vanishing cigarette and lighter (Kuhn & Tatler, 2005)	The magician tries to light a cigarette, but the lighter vanishes and so does the cigarette (Supplemental Movie 9)	The objects are openly dropped in the lap	Attention, inattentional blindness

*Note.* The label for the category of tricks based on attentional and reasoning misdirection is abbreviated to AR.

The tricks were presented in orders following the pattern (X1, Y1, Z1, X2, Y2, Z2, X3, Y3, and Z3), where X, Y, and Z was a randomly chosen order of trick categories (e.g., X = completion, Y = absence, and Z =  “AR”) and the numbers are those in the labels of the tricks (Table 1). Thus, the presentation order of the tricks was fixed within each type of trick, but the relative order of the three different trick categories was randomized. The experiment was conducted with the online survey tool «SurveyXact» (https://www.surveyxact.com). On the first page of the online survey form, the participants received general information about the study (see Appendix A), and online informed consent was obtained by having a final statement at the information page stating that the participant had read the information and was consenting by going further in the survey. Before the main part of the survey commenced, the participants were asked to indicate their age and gender as well as whether they had any experience performing magic, and if so whether it was at an amateur or a professional level. The magic tricks used in the video clips were performed by the first author (M. S.).

Participants were recruited via a Facebook post on the web page of the University of Bergen showing a video of a magic trick and a link to the survey. The post and the survey were in Norwegian. A total of 518 persons clicked the link, but only 75 (46 females and 29 males, with a mean age of 33 and a standard deviation of 13.3) completed the survey. Eleven of the participants reported that they had experience with performing magic. Two of these reported that they had experience performing magic at a professional level. Incomplete data sets from 144 additional participants were not considered further.

The participants written responses regarding how the different magic tricks were performed were coded by author M. S. and a student research assistant who was naive to the purpose and hypotheses of the study. The magic tricks varied in complexity and therefore had one to four theoretically based solution criteria, as described in Appendix B. In the data analysis, we computed solution scores by averaging across the solution criteria. The study was approved by the Norwegian Centre for Research Data (project number 56742).

In a previous similar study (Ekroll, De Bruyckere, et al., 2018), we obtained clear effects with a sample size of 40 participants. Thus, we aimed for at least the same number of participants. A few weeks after we announced the Facebook post it turned out that 75 participants had completed the study, and we regarded that as sufficient to proceed with analysis. All measures, conditions, and data exclusions are reported in the “Results” section.

## Results

### Coding Scheme, Exclusion Criteria, and Preprocessing of Data

The correctness of the participants’ guesses about the secret behind each magic trick was evaluated by author M. S. and a student research assistant who was naive regarding the purpose and hypotheses of the study. Initially, we rated the solutions given by the participant on a 4-point accuracy scale. The interrater agreement was surprisingly low (70%). This made us realize that scoring the correctness of a given response was not quite straightforward for some of the tricks because they involve several critical ingredients. Consider, for instance, the rope trick (“Completion 1,” see Table 1 and Supplemental Movie 4). This trick involves three different instances of amodal completion. Thus, it is not straightforward to decide whether mentioning one, two, or all three should count as a solution of the trick. To address this general problem, we developed a coding scheme with one to four specific criteria to be used for each of the tricks. The coding instructions are listed in Appendix B. The overall interrater agreement pooled across all single criteria was 93% (Cohen’s κ = 0.80), between author M. S. and a new naive rater. For each individual trial, we computed a solution score by averaging the ratings (1 for *correct* and 0 for *not correct*) across both raters and all coding criteria.

Whenever a participant knew the solution of a particular trick in advance, his or her responses to that particular trick was excluded from further analysis. Thus, in 49 of a total of 675 possible cases (7.26%), all three responses of an observer to a given trick were excluded (see Table 2 for a breakdown for the individual tricks). It sometimes happened that a participant stated the right explanation of a given trick after the first or second presentation, but stated another, wrong explanation at a later presentation. In these cases, it is reasonable to assume that the participant already was aware of the right solution but still tried to find alternative solutions. Therefore, whenever a participant’s solution score was lower at a later presentation of a given trick than at a previous presentation of the same trick, we adjusted the later score to match the previous one.

**Table 2.** Number of Participants (Out of 75) Who Knew Each of the Tricks in Advance.

Trick type	Absence			Completion			Attention/reasoning		
Trick variant	1	2	3	1	2	3	1	2	3
Number of participants	5	3	4	7	2	13	3	5	7
%	6.7	4	5.3	9.3	2.7	17.3	4	6.7	9.3

### Solution Scores

**Figure 2.**
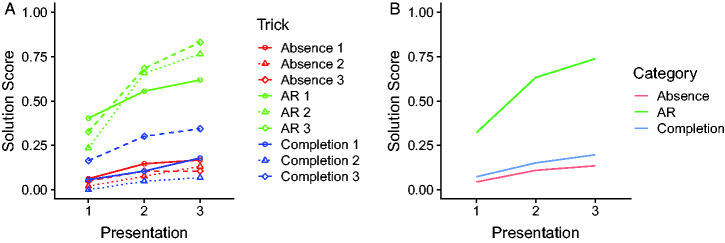
A: Average solution scores for the tricks plotted against how many times they had been viewed. B: Same as (A) but averaged across tricks of the same category. AR = attentional and reasoning.

The average solution scores are plotted against the presentation number in Figure 2. As can be seen, the tricks based on the illusion of absence and amodal completion have the lowest solution scores across all three presentations. To quantify the statistical evidence for differences between the three kinds of tricks in the average solution score, we ran a Bayesian mixed-model analysis of variance (ANOVA) (Morey et al., 2015) for each presentation time and each pair of trick types (absence vs. completion, absence vs. AR, and completion vs. AR) separately. The solution score was the independent variable, trick type was the fixed factor, and subject was entered as a random factor. The resulting Bayes factors are listed in Table 3. The Bayes factors BF_10_ obtained for all comparisons involving the AR tricks are (substantially) larger than 100 and thus provide “decisive” evidence for a difference according to Jeffrey’s terminology (Jarosz & Wiley, 2014, p. 8). Thus, we can be very confident that the solution scores are higher for the AR tricks than any of the other two kinds of tricks. The Bayes factors obtained for the comparisons between tricks based on the illusion of absence and tricks based on amodal completion are smaller than 1 but larger than 1/3 and hence provide “anecdotal” evidence against a difference (Jarosz & Wiley, 2014, p. 8).

**Table 3.** Bayes Factors BF_10_ for Differences in Solution Scores Between Trick Types at Each of the Three Presentation Times.

Comparison	Presentation 1	Presentation 2	Presentation 3
AR versus absence	2.1e + 17	7.6e + 39	3.9e + 52
AR versus completion	5.3e + 11	1.8e + 34	3.2e + 44
Absence versus completion	0.35	0.35	0.96

*Note.* AR = attentional and reasoning.

The results plotted in Figure 2 also show that the change in solution scores from one presentation to the next tends to be larger for the AR tricks than for the other two kinds of tricks, particularly from Presentation 1 to Presentation 2. To quantify the statistical evidence for differences between the three categories of trick with respect to the amount of change with presentation number, we ran a Bayesian mixed-model ANOVAs (Morey et al., 2015) for each pair of presentation times (1–2 and 2–3) and each pair of trick categories (absence vs. completion, absence vs. AR, and completion vs. AR). The solution score was the independent variable, trick type and presentation time were the fixed factors, and subject was entered as a random factor. Table 4 lists the Bayes factors BF_10_ obtained for potential differences in the amount of change (i.e., the interaction between trick category and presentation time). Regarding the changes in solution scores from Presentation 1 to Presentation 2, there is “decisive” evidence (Bayes factors larger than 100) that the change is larger for the AR trick category than for any of the two other trick categories, and “substantial” evidence (a Bayes factor less than 1/3 and larger than 1/10) *against* any difference in that change for the tricks based on amodal absence and those based on amodal completion. From Presentation 2 to Presentation 3, the Bayes factors are all less than 1, meaning that they provide evidence against a difference. But note that the evidence against a difference in the change is only “anecdotal” (between 1/3 and 1) when the AR tricks are compared with the absence tricks. For the other two pairs of trick types, the evidence against a difference is “strong” (less than 1/3). Thus, in summary, we have clear evidence that the solution scores increase more for the AR tricks than the other tricks from Presentation 1 to Presentation 2, but there is no evidence for this from Presentation 2 to Presentation 3.

**Table 4.** Bayes Factors BF_10_ for Differences in the Change in Solution Scores From One Presentation to the Next, for All Three Pairs of Trick Categories.

Comparison	From Presentation 1 to Presentation 2	From Presentation 2 to Presentation 3
AR versus absence	5.2e + 05	0.48
AR versus completion	8.3e + 04	0.25
Absence versus completion	0.12	0.12

*Note.* AR = attentional and reasoning.

### Magic Ratings

**Figure 3.**
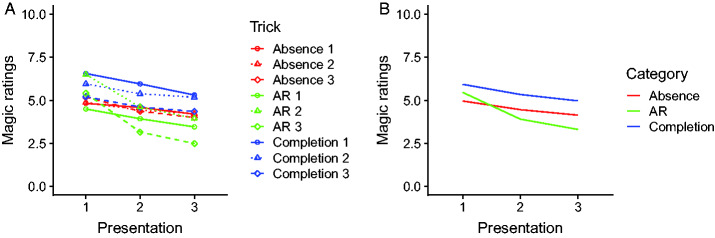
A: Average magic ratings plotted against presentation number, plotted separately for each of the nine tricks investigated. B: Same as in (A) but pooled across all tricks of the same type. AR = attentional and reasoning.

Figure 3 shows the mean magic ratings. The ratings were collected immediately after each presentation of a trick and thus immediately before the participant was asked to report what she or he thought may be the secret behind the trick. Panel A shows the mean ratings for each trick separately, at each of the three presentations. Panel B shows the same data averaged across tricks of the same category. As can be seen in (A), the magic ratings decrease with repeated presentations for all the individual tricks. The slope of the decrease is similar for most of the tricks, except two of the AR tricks, where the drop from Presentation 1 to Presentation 2 is somewhat steeper. On average, the tricks based on amodal completion have the highest ratings, those based on amodal absence have intermediate ratings, and the AR tricks have the lowest ratings. The decrease in the magic ratings with presentation number is very similar for the tricks based on amodal completion and the tricks based on the illusion of absence but somewhat larger for the AR tricks (Figure 3B). A Bayesian mixed-model ANOVA (Morey et al., 2015) with the magic ratings as the independent variable, trick category, and presentation number as fixed factors and subject as a random factor revealed “decisive evidence” for a main effect of trick category (BF_10_ = 2.4e + 15), for a main effect of presentation number (BF_10_ = 4.4e + 24), as well as for an interaction between these two factors (BF_10_ = 1,265). We also ran a Bayesian mixed-model ANOVA for each presentation time and each pair of trick types (absence vs. completion, absence vs. AR and completion vs. AR) separately. The magic rating was the independent variable, trick type was the fixed factor, and subject was entered as a random factor. The resulting Bayes factors are listed in Table 5. There was “decisive” evidence for the differences between the tricks based on amodal completion and those based on the illusion of absence at all presentation times (Bayes factors larger than 100). There was also “decisive” evidence for a difference between the AR tricks and the amodal completion tricks at Presentation 2 and Presentation 3, but at Presentation 1, there was “anecdotal” evidence against a difference. Finally, there was evidence for a difference between the AR tricks and those based on the illusion of absence at all presentations, but only “anecdotal” at Presentation 1, “substantial” at Presentation 2, and “decisive” at Presentation 3.

**Table 5.** Bayes Factors BF_10_ for Differences in Magic Ratings Between Trick Types at Each of the Three Presentation Times.

Comparison	Presentation 1	Presentation 2	Presentation 3
AR versus absence	2.82	3.68	720
AR versus completion	0.74	1.6e + 7	8.6e + 9
Absence versus completion	2,200	335	111

*Note.* AR = attentional and reasoning.

To quantify the statistical evidence for differences between the three categories of trick with respect to the amount of change with presentation number, we ran a Bayesian mixed-model ANOVAs (Morey et al., 2015) for each pair of presentation times (1–2 and 2–3) and each pair of trick categories (absence vs. completion, absence vs. AR, and completion vs. AR). The magic rating was the independent variable, trick type and presentation time were the fixed factors, and subject was entered as a random factor. Table 6 lists the Bayes factors BF_10_ obtained for potential differences in the amount of change (i.e., the interaction between trick category and presentation time). From Presentation 1 to Presentation 2, there is evidence that the change is larger for the AR tricks than for either of the two other kinds of tricks. This evidence is “decisive” (BF_10_ > 100) for the comparison with the tricks based on the illusion of absence “very strong” (BF_10_ > 30) for the comparison with the tricks based on amodal completion. In all other cases, there is “substantial” evidence against different amounts of change (BF_10_ < 1/3).

**Table 6.** Bayes Factors BF_10_ for Differences in the Change in Magic Ratings From One Presentation to the Next, for All Three Pairs of Trick Categories.

Comparison	From Presentation 1 to Presentation 2	From Presentation 2 to Presentation 3
AR vs. absence	143.5	0.17
AR vs. completion	37.8	0.14
Absence vs. completion	0.11	0.11

*Note.* AR = attentional and reasoning.

### Relationship Between the Solution Scores and the Magic Ratings

**Figure 4.**
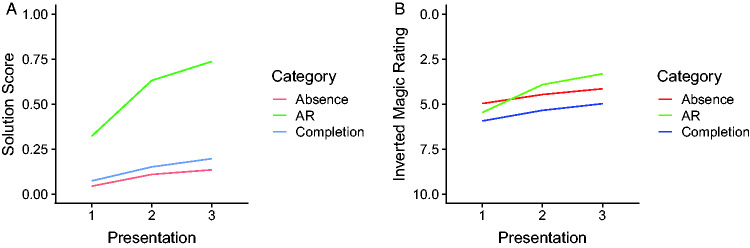
The average solution scores (A) and the average magic ratings (B) shown next to each other. To aid comparison, the magic ratings are plotted on an inverted axis, with the lowest possible magic rating (0) on the top and the highest possible rating (10) on the bottom. AR = attentional and reasoning.

Intuitively, it seems reasonable to expect an inverse relationship between solution scores and magic ratings because knowing the secret behind a trick can be expected to make it appear less magical. On this view, one would expect that the pattern of solution scores in Figure 2B should be qualitatively similar to the pattern of ratings in Figure 3B except for an inversion along the vertical axis. In Figure 4, these two patterns of results are shown next to each other using an inverted axis for the magic ratings. As can be seen by comparing the two figures, there are indeed some similarities, but there are also differences. A common feature (that is also documented by the statistical analyses in Tables 4 and 6) is that the AR tricks change more than the two other kinds of tricks from Presentation 1 to Presentation 2. A further common feature is that the overall average is higher for the AR tricks than for the two other categories of tricks. A difference that is apparent by comparing the two plots, however, is that the direction of the difference between the tricks based on the illusion of absence and those based on amodal completion is reversed. This contradiction is not necessarily as blatant as it may seem, though, given that the statistical evidence suggests that the observed differences in solution scores may be spurious (Bayes factors less than 1, see Table 3, bottom row). A further notable difference is that the range across which the average magic ratings vary is rather small (Figure 4B), while the corresponding range for the average solution scores is quite substantial (Figure 4A).

**Figure 5.**
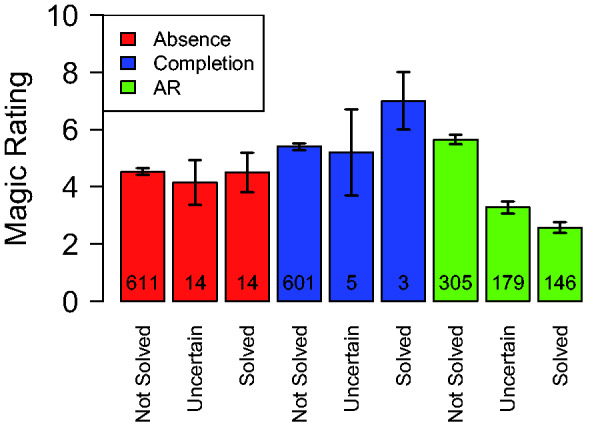
Average Magic Ratings for Each of the Three Kinds of Tricks, Plotted Separately Depending on Solution Status. The numbers of observations underlying the averages are printed on the bars. The error bars indicate one standard error of the mean in each direction. AR = attentional and reasoning.

Figure 5 shows the relationship between solution scores and magic ratings in a different format.

Here, the magic ratings are averaged within each category of trick and across presentation times but separately for three different types if trials. In the first type of trial (“not solved”), the participant had not yet solved the trick. In the second type of trial (“uncertain”), the participant solved the trick in the same trial as the magic rating was given, but as the magic rating was made at the beginning of the trial, it is uncertain whether the subject knew the solution while making the rating or not. In the third type of trial (“solved”), the participant had solved the tricks in the previous trial so that we can be certain that the participant was aware of the solution when the magic rating was made. We categorized a trial as solved only when the solution score equaled 1. A curious pattern that emerges in Figure 5 is that the solved trials do not have lower average magic ratings than the unsolved trials for all trick categories. Only the AR tricks exhibit this intuitively expected pattern. Furthermore, even though the AR tricks do exhibit the expected decrease in magic ratings when the tricks are solved, the average magic rating is far from zero in the trials where the tricks had already been solved. Note that the unexpected patterns of results obtained for the tricks based on amodal completion and those based on amodal absence are based on relatively few data points simply because those tricks were infrequently solved (as already apparent in Figure 2). In Figure 5, the numbers of relevant observations are plotted on the corresponding bars. Statistical analysis, however, indicate that there is “substantial” statistical evidence against a dependence on solution status both for the tricks based on the illusion of absence (BF_10_ = 0.15) and for the tricks based on amodal completion (BF_10_ = 0.30). For the AR tricks, on the other hand, there was “decisive” evidence that the magic ratings do depend on the solution status (BF_10_ = 2.4e + 36).

**Figure 6.**
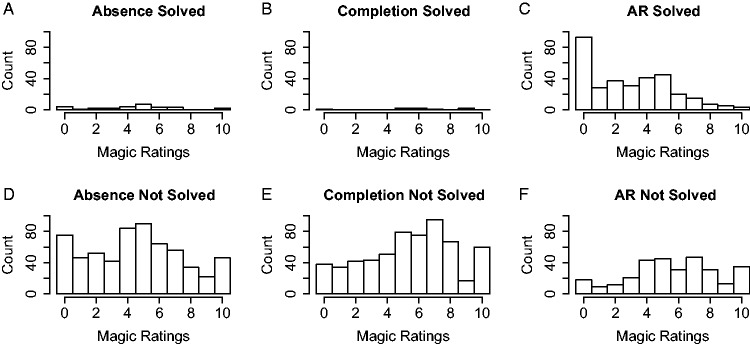
Histograms of the Magic Ratings for Each of the Three Types of Trick, Plotted Separately for Trials Where the Trick Was Solved (Top Row) and for Trials Where the Trick Was Not Solved (Bottom Row). Notice how broad the distributions are, that magic ratings of 0 occurred even when the trick was not solved, and that magic ratings of 10 occurred even when the trick was solved. AR = attentional and reasoning.

Figure 6 shows the distributions of magic ratings underlying the average values shown in Figure 5. Note that the distributions are rather broad, meaning that the magic ratings vary substantially, both when the tricks were solved in given trial and when they were not. Interestingly, the distributions are even so broad that there were (A) cases where participants gave a magic rating of 10 even when they correctly solved the trick in the corresponding trial, as well as (B) cases where the participants gave a magic rating of 0 although they had failed to solve the trick.

**Figure 7.**
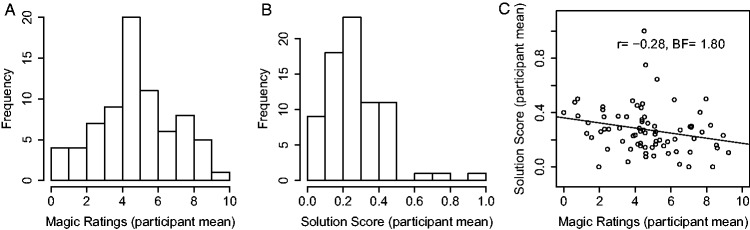
A: Distribution of participant-specific overall magic ratings (averaged across all tricks and presentation numbers). B: Distribution of participant-specific overall solution scores. BF = Bayes factor.

Figure 7 shows the distributions of the average magic rating of each participant (Panel A) and the average solution score of each participant (Panel B). While the distribution of the individual average magic rating levels peaks at intermediate values (between 4 and 5), there are also participants who generally tend to give extremely low (between 0 and 1) or extremely high (between 9 and 10) magic ratings. The distribution of the individual solution scores, on the other hand, peaks at a low value (between 0.2 and 0.3), and most of the participants have an individual solution score well below 0.5. Figure 7C plots the individual average magic ratings against the individual solution scores. On the descriptive level, there is a small negative Spearman correlation (*r* = −.28), but the statistical evidence for a nonzero correlation is merely “anecdotal” (BF_10_ = 1.80). Thus, the individual tendency to give high or low magic ratings is at best only marginally related to the individual problem-solving performance.

## Discussion

Our results show that very few subjects figured out how the magic tricks based on the illusion of absence and the magic tricks based on amodal completion were done after repeated presentations, while the majority did so for magic tricks based on attentional and reasoning misdirection. Such a difference between tricks based on amodal completion and tricks based on attentional misdirection has already been observed in a previous study (Ekroll, De Bruyckere, et al., 2018). In addition to replicating this previous finding, the present findings indicate that tricks based on the illusion of absence are similarly robust to repetition as tricks based on amodal completion. As previously argued by Ekroll, De Bruyckere, et al. (2018), tricks based on perceptual illusions can be expected to be robust to repetition due to the robust and persistent nature of perceptual illusions (Firestone & Scholl, 2016; Leslie, 1988; Pylyshyn, 1999). Thus, the present findings suggest that the mechanisms underlying the illusion of absence are perceptual in nature, just like those underlying amodal completion (Ekroll, Mertens, et al., 2018; Ekroll & Wagemans, 2016; Gerbino & Zabai, 2003; Kanizsa, 1985; Michotte et al., 1964; Scherzer & Ekroll, 2015; Shimojo & Nakayama, 1990).

### Visual Perception of Occluded Space

It may certainly appear counterintuitive to claim that perceptual or visual mechanisms determine our experience of regions of space which are occluded from direct view and hence produce no visual stimulation whatsoever. It is already well known, however, that perceptual processes mostly do not have direct access to the real-world properties they make inferences about and instead rely on indirect cues and contextual information (Gilchrist, 2015; Hoffman, 2000; Palmer, 1999). The perceptual experience of three-dimensional depth, for instance, is certainly not a product of direct sensory stimulation (as the retina is two-dimensional rather than three-dimensional), but rather a product of indirect cues, higher order regularities, and contextual information that actually are available in the more global pattern of sensory stimulation at the retina (Hershenson, 1999). Thus, the claim that visual mechanisms can determine our experience of occluded regions of space is, in general principle, no more radical than the commonly accepted view that our experience of depth is determined by visual mechanisms. There is certainly contextual information in the stimulus that sometimes makes it possible to make educated guesses about what may or may not be hidden behind an occluder, and the interesting empirical question is to what extent and according to what rules and heuristics the visual system actually uses this information to make such “educated guesses.” A large body of research on amodal completion already shows that, although it may appear counterintuitive, the visual system uses such information to an impressive extent (Ekroll, Mertens, et al., 2018; Ekroll et al., 2017; Gerbino, 2017; Gerbino & Zabai, 2003; Kanizsa, 1985; Koenderink et al., 2018; Michotte et al., 1964; Peta et al., 2019; Scherzer & Ekroll, 2015; Shimojo & Nakayama, 1990; Tse, 1999; Van Lier, 1999; Van Lier & Wagemans, 1999). The present findings suggest in agreement with the findings of Øhrn et al. (2019) that the visual system also can create a perceptual illusion of absence by relying on contextual information. Importantly, extant models of amodal completion which appeal to the notion that visible parts form the basis for some kind of perceptual inter- or extrapolation of the invisible parts cannot be applied to the illusion of absence because it does not involve any visible parts. The generic view principle (Albert, 2001; Albert & Hoffman, 2000), however, seems to furnish a plausible candidate explanation and the results of Øhrn et al. (2019) provide some preliminary support for this hypothesis. A prediction that can be derived from this hypothesis is that the illusion of absence should be stronger or more likely to occur for narrow occluders than wide occluders, which was indeed found in Øhrn et al.’s study, although the difference was rather small. This hypothesis needs to be tested further. It would also be desirable to develop alternative candidate explanations for the illusion of absence.

### Weak Relationship Between Solution Scores and Magic Ratings

Several aspects of our data show that the relationship between the solution scores and the magic ratings deviate considerably from what one might expect based on the simple notion that the trick should be rated as magical if and only if the spectator is unaware of the secret behind it:

1. The overall solution scores for the occlusion tricks (i.e., those based on the illusion of absence or amodal completion) are much higher than the overall solution scores for the AR tricks (Figure 2B), while the corresponding difference in overall magic ratings is rather small (Figure 4B).
2. Relatedly, the difference between the solution scores of the occlusion tricks, on the one hand, and the AR tricks, on the other hand, increases considerably with repeated presentations (Figure 2B), but the corresponding difference in magic ratings increase only marginally (Figure 4B).
3. The average magic ratings were not very different for trials where the participants did not know the secret behind the trick and trials where they definitely did (Figure 5). Indeed, for the occlusion tricks, there was “substantial” statistical evidence *against* a dependence on solution status.
4. Even for the AR tricks, which exhibited the intuitively expected decrease in magic ratings for solved trials, the average magic rating in the solved trials was still considerably above zero.
5. The correlation between the individual general propensities to solve the tricks and the individual general propensities to give high magic ratings was small (*r* = −.28, Figure 7) and only supported by “anecdotal” statistical evidence.

Two general hypotheses may together furnish a plausible account for the rather high magic ratings in the trials where the participants had already figured out the secret behind the trick, namely, (a) that the discovery of the secret behind the trick also evokes an experience of magic and (b) that tricks based on cognitively impenetrable perceptual illusions may retain a certain residual magical quality even after the secret is known because perceptual illusion tend to persist even in the face of better knowledge (Ekroll et al., 2017; Ortiz, 2006).

#### Magical Experiences Evoked by Discovering the Secret Behind a Trick

At first blush, one might be prone to think that discovering the secret behind a trick is a disenchanting and sobering experience. Several lines of reasoning, however, suggest that it may actually evoke feelings of surprise, insight, and perhaps even an illusion of impossibility. Discovering the secret behind a magic trick often evokes an Aha-experience or experience of insight (Danek, 2018; Danek & Flanagin, 2019; Danek et al., 2014, 2018). As discussed by Topolinski and Reber (2010), two of the main characteristics of insight are that the “solution of the problem pops into mind abruptly and surprisingly” (p. 402) and that the insight “yields a genuine positive affective experience” (p. 402). Surprise is rated as one of the more favored aspects of magic tricks (Gronchi et al., 2017; Jay, 2016). Thus, the rather high magic ratings made by the participants in our study after they had discovered the secret may reflect the surprise they experienced when realizing how simple the secrets behind the originally rather impressive magic tricks turned out to be. Furthermore, it may be that discovering the secret behind a magic trick actually evokes a magical experience in the sense that it leads to an illusion of impossibility (Aronson, 2013). In many magic tricks, the secret is so simple and blatantly obvious in retrospect, that once the spectator knows the secret, they may find it incomprehensible how they could be fooled by the trick in the first place. A key reason why it may be difficult to understand why one is so easily fooled by many magic tricks is that they are based on systematic failures in visual metacognition (Ekroll, 2019; Ekroll et al., 2017; Kuhn, 2019; Kuhn et al., 2014; Ortega et al., 2018). These failures of metacognition are highly counterintuitive and have consequences that may appear impossible (for instance, that you failed to notice something that happened right in front of your eyes or that your brain made you “hallucinate” missing pieces of an object). The broad public appeal of phenomena like change blindness may stem from the fact that they involve highly counterintuitive and surprising failures of visual metacognition (Simons & Rensink, 2005; Levin, 2002). Thus, one might speculate that both demonstrations of change blindness and discovering the secret behind magic tricks may evoke magical experiences for essentially the same reason: The associated failures of visual metacognition have consequences that appear impossible.

#### Residual Magical Qualities of Magic Tricks Based on Perceptual Illusions

As pointed out by Ekroll et al. (2017), tricks based on visual illusions may be expected to retain a residual “magical quality” (p. 94) even after the secret has become known. Although the spectator knows that what happens is not really impossible, it still looks like something impossible happens because the underlying visual illusion persists in spite of better knowledge (Firestone & Scholl, 2016; Kanizsa, 1985; Leslie, 1988).

On their own, neither of the two above hypotheses can explain the observed pattern of results, but together they furnish a plausible account. The hypothesis that discovering the secret behind a trick can evoke a magical experience explain why the magic ratings are relatively high after discovery of the secret for all three types of tricks (Figure 5), and the hypothesis that tricks based on perceptual illusions lead to a residual magical quality even after discovery of the secret can explain why the magic ratings after discovery tend to be somewhat higher for the tricks based on amodal completion and the illusion of absence (Figure 5). The former hypothesis is entirely ad hoc, but we think is theoretically interesting and warrants further investigation.

### Criteria for Having Discovered the Secret(s) Behind a Magic Trick

One challenge we faced in this study was how to best rate whether the participants had solved the magic tricks or not. As mentioned, the responses were initially scored on a 4-point rating scale, similar to that used by earlier studies in which magic tricks were used as insight problems (Hedne et al., 2016). In our case, this way of determining the accuracy of the responses gave a surprisingly low interrater agreement (70%). In the Hedne et al.’s (2016) study, both raters were professional magicians with a deep understanding of the workings behind the tricks, while in this study, only one of the raters had experience with performing magic. This might suggest that using atheoretical ratings as a measure of insight into the secret behind magic tricks requires raters with a relatively deep knowledge of the magic techniques used in the tricks. In hindsight, we should have selected only tricks with a single theoretically relevant solution criterion. Such tricks would presumably be easier to rate, especially for naive raters without any prior knowledge of magic.

A limitation of the web-based survey application we used was that it did not let us control how many times each participant viewed the videos, apart from instructing them not to. Thus, we cannot rule out that some participants may have seen the tricks more times than intended. If this happened it may have given us some false positives.

All the magic tricks used in the illusion of absence category relied on the magic technique of *palming* (de Ascanio, 1964/2005), which is a collective term for sleight of hand techniques involving secretly hiding an object in an apparently empty hand. The technique is used in a variety of magic tricks in order to create the illusion that objects magically appear or disappear. Our results indicate that the illusion of absence is indeed a perceptual illusion, and it appears likely that this perceptual illusion plays a pivotal role in making palming such a powerful magical technique. An interesting informal observation we made when selecting tricks for this study was that we found it rather difficult to find tricks for the amodal completion and AR categories that did not involve a potential additional contribution of the illusion of absence due to palming or other secret moves where objects are completely hidden. This suggest that the illusion of absence might play a very pervasive role in magic and thus be a key perceptual process in the magician’s toolbox.

## Conclusions

In agreement with the findings of Øhrn et al. (2019), the present results lend further support to the hypothesis that the illusion of absence is based on cognitively impenetrable perceptual mechanisms in much the same way as classical amodal completion. Our findings also suggest that there may be two magical moments in the lifetime of a magic trick: In addition to the magical experience evoked by trick itself, discovering the secret behind the trick may also evoke an experience of impossibility. The latter hypothesis is ad hoc but appears well worth pursuing in further research.

## Appendix A: General Information About the Study Given to the Participants at the Beginning of the Experiment (English Translation of the Norwegian Original)

Welcome. Thank you for participating in this experiment. You are about to watch some magic tricks. You will rate how magical you think they are on a scale of 0 to 10. You shall also enter how you think the magician did the trick. The purpose of the study is to study how visual processes play a role in magic tricks. Participation is voluntary and you can withdraw at any time during the survey. Your participation is anonymous. The study is conducted at the University of Bergen. The responsible for the study are Prof. Vebjørn Ekroll at the Department of Social Psychology, Vebjorn.Ekroll@uib.no, and Mats Svalebjørg, msv009@student.uib.no. The project is approved by the Norwegian Centre for Research Data. As I click “next,” I confirm I have read and understood this text and give my consent for participation.

### Appendix B: Criteria for Coding the Solutions (English Translation of the Norwegian Original)

The following is an overview of the solutions of the magic tricks used in the study and the criteria the solution was scored by. The magic tricks vary in complexity, where some tricks only have one criterion for a complete solution, while others have multiple criterions.

#### Amodal Absence

##### Absence 1: Production of Jumbo Coin From Purse

This magic trick is done by the coin being hidden at first in the left hand behind the purse when the right hand is displayed being empty. Then the coin is then transferred along with the purse to the right hand as the left hand is displayed being empty. The left hand grabs the purse and the right hand that now conceals the coin simulates taking the coin out of the purse.

There are four key criteria in the magic trick:

The coin was in the left hand.

The coin was in the right hand.

The coin was transferred with the wallet.

The simulation of taking the coin out from the purse.

##### Absence 2: Disappearing Cigarette

This magic trick is done by having the cigarette being secretly transferred to the left hand after it apparently is being pushed into the right hand. In the left hand, it is kept hidden between the middle finger and the palm of the hand, going parallel to the middle finger.

If the participant describes where the cigarette ends up, the trick is scored as being solved.

##### Absence 3: Vanishing Cards

In this magic trick, the cards that are apparently being dealt out of the right hand is collected in the hand as they «vanish». All cards are collected in a pile in the right hand during the deal before they are dropped into the lap at the end.

If the participant understands that the cards are collected together in the hand, they have solved the trick.

#### Amodal Completion

##### Completion 1: Rope Trick

This trick is done using two ropes, a short and a long rope. First, the two ropes are shown as one rope. When the ends are picked up with the right hand, both ends of the long rope are caught in the left hand while both ends of the short rope are visible. Then, the magician simulates taking the ends off the rope. Then, it is simulated that the long rope is a closed loop. Finally, the little rope is thrown back and one of the ends of the long rope is released as the magician catches one end of the short rope, again showing the two ropes as one.

There are three key criteria in the trick:

The two ropes are held together as one in the beginning.

The long rope is shown as a closed loop.

The short rope goes back to the long rope, reaching the starting position.

##### Completion 2: Pencil Through Banknote

This trick is done with an extra piece of plastic on the pencil. This piece achieves the illusion that the pencil is inside the banknote while it’s really behind.

If the participant describes something that enables this illusion, the trick is solved.

##### Completion 3: Linking Rings

In this trick a closed metal ring and a metal ring with an opening are used. The ring in the left hand is the one with the opening. When the rings are shown as whole, the fingers on the left hand covers the opening the whole time. The rings are then hooked together. If the participant describes that there is an opening that is always concealed the trick is solved.

There are three criteria for judging this:

One ring has an opening.

The opening is constant, there is no hidden mechanisms that opens up temporarily.

The opening is hidden in the hands.

#### Attentional and Reasoning Misdirection

##### AR 1: Disappearing Ball

This magic trick is done by dropping one ball behind the table when the two balls are apparently put together.

If the participant describes that the ball is dropped behind the table, the trick is solved.

##### AR 2: Coin Under Card

This trick is done by flicking the coin under the card when it simulated that the coin is picked up by the left hand.

If the participant describes that the coins are pushed under the card at that moment, they have solved the trick.

##### AR 3: Vanishing Cigarette and Lighter

This trick is done by dropping both the cigarette and the lighter openly behind the table.

There are two key criteria in the magic trick:

The dropping of the cigarette.

The dropping of the lighter.
